# Comparative antler proteome of sika deer from different developmental stages

**DOI:** 10.1038/s41598-021-89829-6

**Published:** 2021-05-18

**Authors:** Ranran Zhang, Yang Li, Xiumei Xing

**Affiliations:** Institute of Special Animal and Plant Sciences, Chinese Academy of Agricultural Sciences, Changchun, China

**Keywords:** Bone development, Gene expression

## Abstract

Antler is a special bone tissue that has the ability to regenerate completely periodically. It is the fastest growing bone in the animal kingdom. Antler provides a valuable research model for bone growth and mineralization. Antler grows longitudinally by endochondral ossification with their growth center located in its tip. Many scholars have carried out detailed studies on morphology and gene expression of antler tip. However, few scholars have analyzed the protein expression patterns of antler tip at different development stages. This study used label-free proteomics approach to analyze the protein expression dynamics of the antler tip in six developmental periods (15, 25, 45, 65, 100 and 130 days after the previous antler cast) and costal cartilage. In result, 2052 proteins were confidently quantified, including 1937 antler proteins and 1044 costal cartilage proteins. Moreover, 913 antler core proteins and 132 antler-special proteins were obtained. Besides, the stages special proteins and differentially expressed proteins (DEPs) in different development stages were analyzed. A total of 875 DEPs were determined by one-way AVOVA. It is found that the growth period (15, 25, 45 and 65 days) showed more up-regulated protein including several chondrogenesis-associated proteins (collagen types II, collagen types XI, HAPLN1, PAPSS1 and PAPSS2). In ossification stages, the up-regulated proteins related with lysosome (CTSD, CTSB, MMP9, CAII) indicated that the antler has higher bone remodeling activity. Given the up-regulated expression of immune-related molecules (S100A7, CATHL7, LTF, AZU1, ELANE and MPO), we speculate that the local immune system may contribute to the ossification of antler tip. In conclusion, proteomics technology was used to deeply analyze the protein expression patterns of antler at different development stages. This provides a strong support for the research on the molecular regulation mechanism of rapid growth and ossification of velvet antler.

## Introduction

There are different types of bones with different characteristics^1^. John Currey strongly advocated studying bone tissues that were not familiar in order to broaden the understanding of the structure–function relationship of vertebrate bones^2^. He was interested on the study of antlers due to their special features. Antler is a head appendage of *Cervidae* different from other ruminants. Antler can regenerate completely periodically. Antler is the fastest growing bone in the animal kingdom. This makes antler a good model for studying bone growth. Currently, extensive research has been carried out on the regeneration and development mechanism of antler and substantial progress has been made. Morphology, histology and experiments have proved that the regenerated tissue that forms the antler blastema is derived from the pedicle periosteum (PP). PP is a derivative of the antler antlerogenic periosteum (AP) which is responsible for the initial pedicle and first antler formation^3–5^. Studies from different laboratories have shown that cells from AP and PP have stem cell properties. Therefore, velvet regeneration is considered to be a regeneration process based on stem cells^6^.

The growth and ossification of antler is different from that of long bones. Growth plates and secondary ossification centers are the characteristics of mammalian long bone growth but they are absent in growing antlers. The growth of antlers is a manifestation of cartilage ossification which involves endochondral ossification and intramembranous ossification. Each antler branch extends longitudinally through endochondral ossification. Moreover, the growth of bone around the antler shaft increases its diameter through intramembranous ossification^7^. Type II collagen is a marker of chondroprogenitor. Price et al. demonstrated the presence of type II collagen at the tip of antler by in situ nucleic acid hybridization and immunocytochemistry^8^.

The antler growth center is located at the tip. The antler growth is similar to epiphyseal growth plate in some aspects. The antler tip is a typical far and near zone and the transition is gradual. However, the boundary between different regions is complicated. Histologically, the antler tip was divided into five tissue layers from distal to proximal: reserve mesenchyme, pre-cartilage, transition zone, cartilage and mineralized cartilage. Ba et al. analyzed the gene expression patterns of these five layers and found the hub genes and signaling pathway related to chondrogenesis and osteogenesis^9^. Through RNA-seq methods, gene expression patterns of the skin, mesenchyme, pre-cartilage and cartilage also were analyzed and series of genes that contributed to chondrogenesis were identified^10^.

Antler usually takes 4–5 months with its longitudinal growth showing a typical S-shaped curve. Growth starts slowly in the spring and accelerates exponentially during the summer. During the growth phase, elongation of antlers in some large deer species (wapiti or moose) can exceed 2 cm/day. This represents the fastest tissue growth rate in the animal kingdom^11^. Elongation slows while testosterone rise as the days shorten in late summer. Moreover, the antlers become heavily mineralized while velvet is shed in preparation for the autumn mating season. The biological characteristics of rapid growth and ossification provide a valuable research model for bone growth and mineralization. To investigate the molecular mechanism, Zhao et al. performed gene expression analysis for 15-day, 60-day and 90-day antler based on the RNA-seq approach. These represented slow growth stage, rapid growth stage, and ossification stages respectively^12,13^.

Protein participates in various biological processes as the direct executor of gene function. Studies have shown that since transcripts fails to consider various post-translational modifications. Moreover, transcripts are only a moderate predictor of protein expression. Therefore, changes in RNA levels cannot completely determine changes in protein levels. Currently, label-free proteomics technology has been widely used in the study of biological development. This provides a more comprehensive and accurate understanding of biological development^14,15^. At present, the whole-genome sequences of sika deer (*Cervus nippon*) was assembled and deposited in the Genome Warehouse in the National Genomics Data Center, Beijing Institute of Genomics (China National Center for Bioinformation), Chinese Academy of Sciences, under accession number GWHANOY00000000, which is publicly accessible at https://bigd.big.ac.cn/gwh. This provided strong data support for the mining of key genes and construction of network regulation for the rapid growth and ossification of antler.

In this study, based on the label-free proteomics technology, an in-depth analysis of the protein expression changes of sika deer antler was conducted in six different developmental periods across slow growth stages (15 and 25 days), rapid growth stages (45 and 65 days) and ossification stages (100 and 130 days). In total, 2052 proteins were confidently quantified, including 1937 sika deer antler proteins and 1044 sika deer costal cartilage proteins. A total of 132 antler-specific proteins were successfully obtained as compared with sika costal cartilage. 875 differentially expressed proteins were screened using one ANOVA method. By analyzing the expression trend of these proteins in different biological periods of antler, we found that the main characteristic of antler growth period may be chondrogenesis, while the ossification stage is mainly the reconstruction of bone matrix. Moreover, we speculate that the local immune system may contribute to the ossification of antler tip. In conclusion, the protein expression dynamics during the regeneration cycle of sika deer antler was described. This was beneficial in determining the rapid growth and ossification mechanisms of antler.

## Result

### Protein identification based on label-free

**Figure 1:**
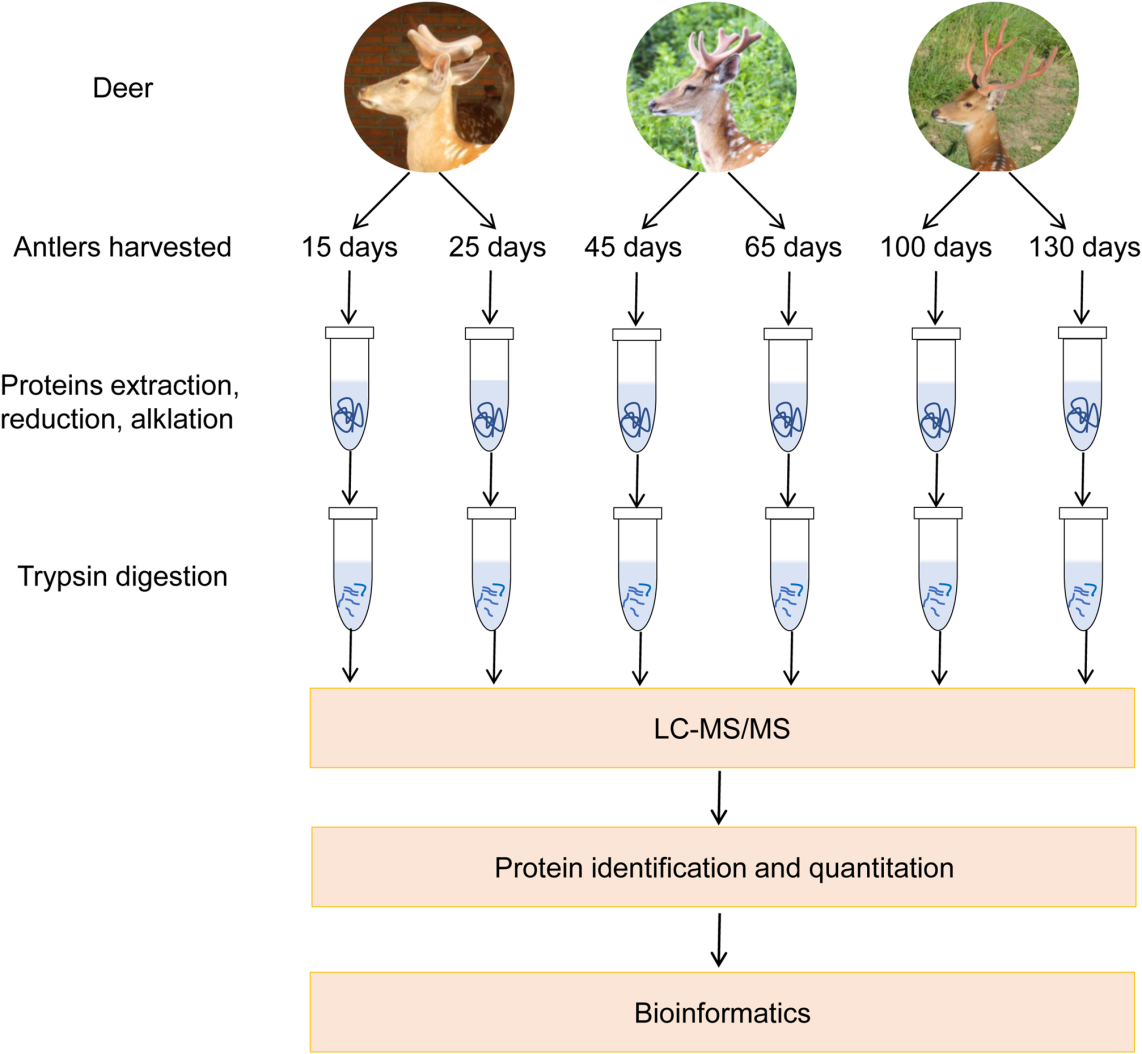
Flowchart of proteomic analysis of sika deer antlers at different development stages. 3 4-year-old male sika deer were selected. The two antlers from first deer were harvested at 15 and 25 days respectively, the two antlers of the second deer were harvested at 45 and 65 days respectively, and the two antlers of the last deer were harvested at 100 and 130 days respectively. LC–MS/MS was performed three times per sample.

**Figure 2:**
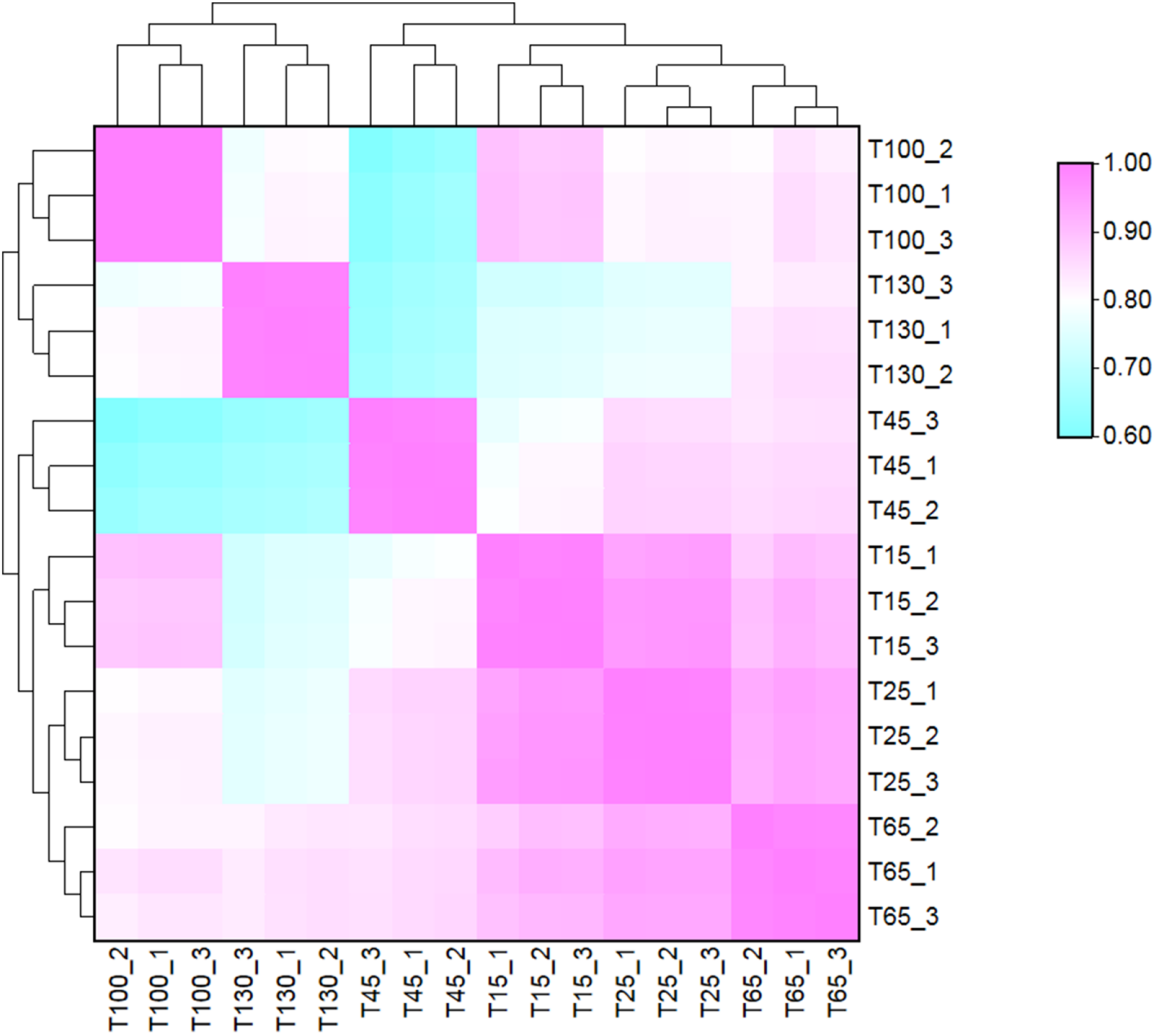
Pearson correlation analysis of sika deer antlers in 6 different development stages.

**Figure 3:**
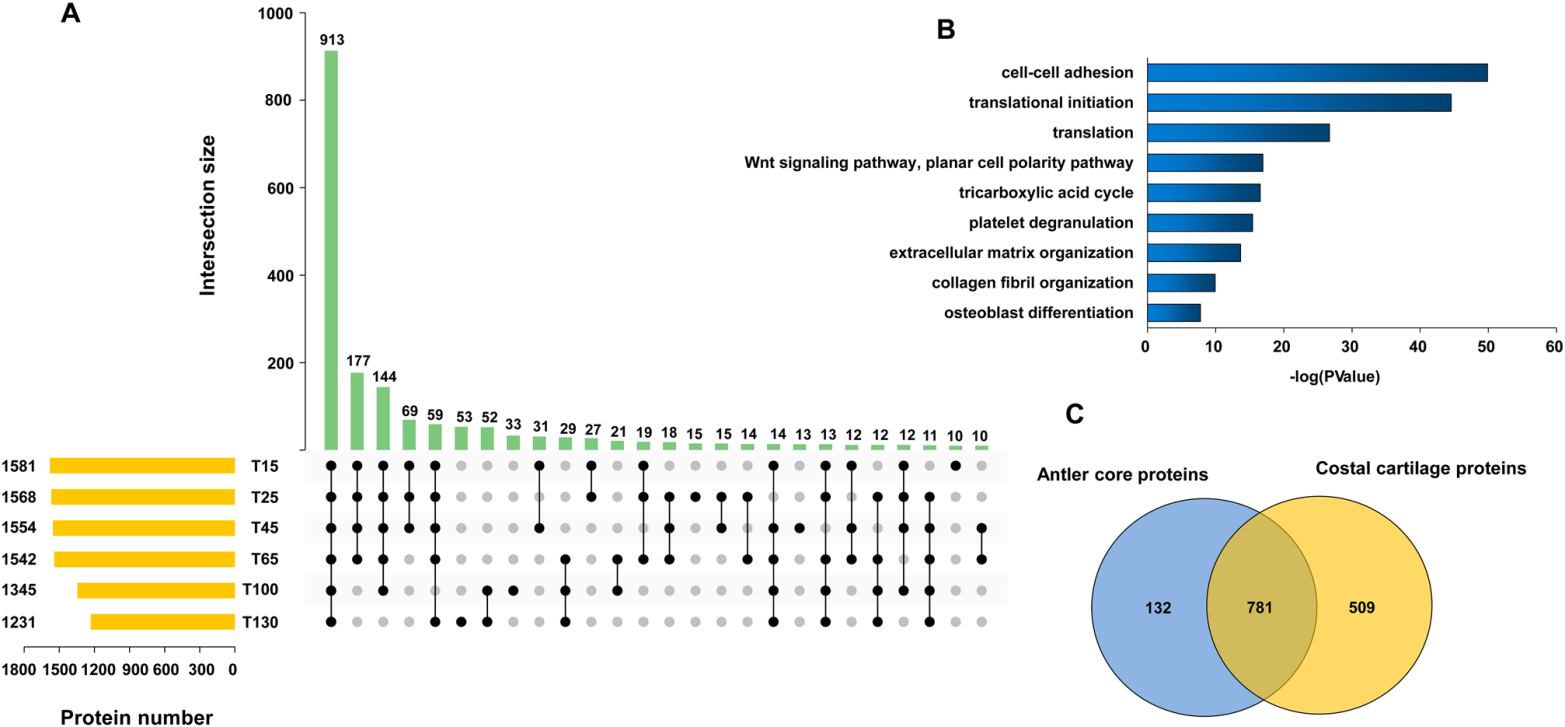
Overview of proteins identified for antler in different development stages and costal cartilage. (**A**) The upset plot diagram showing overlapping proteins identified from antlers in six different development stages. (**B**) The top nine terms of GO enrichment result for 913 antler core proteins. (**C**) The Venn diagram showing overlapping between antler core proteins and costal cartilage proteins.

In this study, antlers from 6 representative time points (Fig. 1) and costal cartilage were collected and LC–MS/MS was performed three times per sample. The mass spectrometry proteomics data have been deposited to the ProteomeXchange Consortium via the PRIDE partner repository with the dataset identifier PXD024323 (https://www.ebi.ac.uk/pride/). Based on the sika deer protein sequences database, 3218 protein groups with 35,379 unique peptides at FDR < 0.01 were identified. Robust detection was filtered in at least two replicates of any sample. In result, we obtained 2052 proteins, including 1937 antler protein and 1044 costal cartilage proteins (Table S1). Pearson correlation analysis was performed to evaluate the MS/MS data (Fig. 2, Table S2). The Pearson correlation coefficients of 3 LC–MS/MS repetitions ranged from 0.989 to 0.999. This indicated high technical reproducibility of the mass spectrometer. In addition, the antler of 15 days, 25 days, 45 days, and 65 days had a high correlation (R = 0.836–0.967). However, the correlation with the antler of 100 days and 130 days was relatively low (R = 0.606–0.853). Sample cluster analysis yielded similar results. The first four periods were clustered into one category and then clustered within 100 days and 130 days. Moreover, the amounts of proteins in the first four periods were similar while the latter two periods were relatively small (Fig. 3A).

### Core proteins for sika deer antler

**Figure 4:**
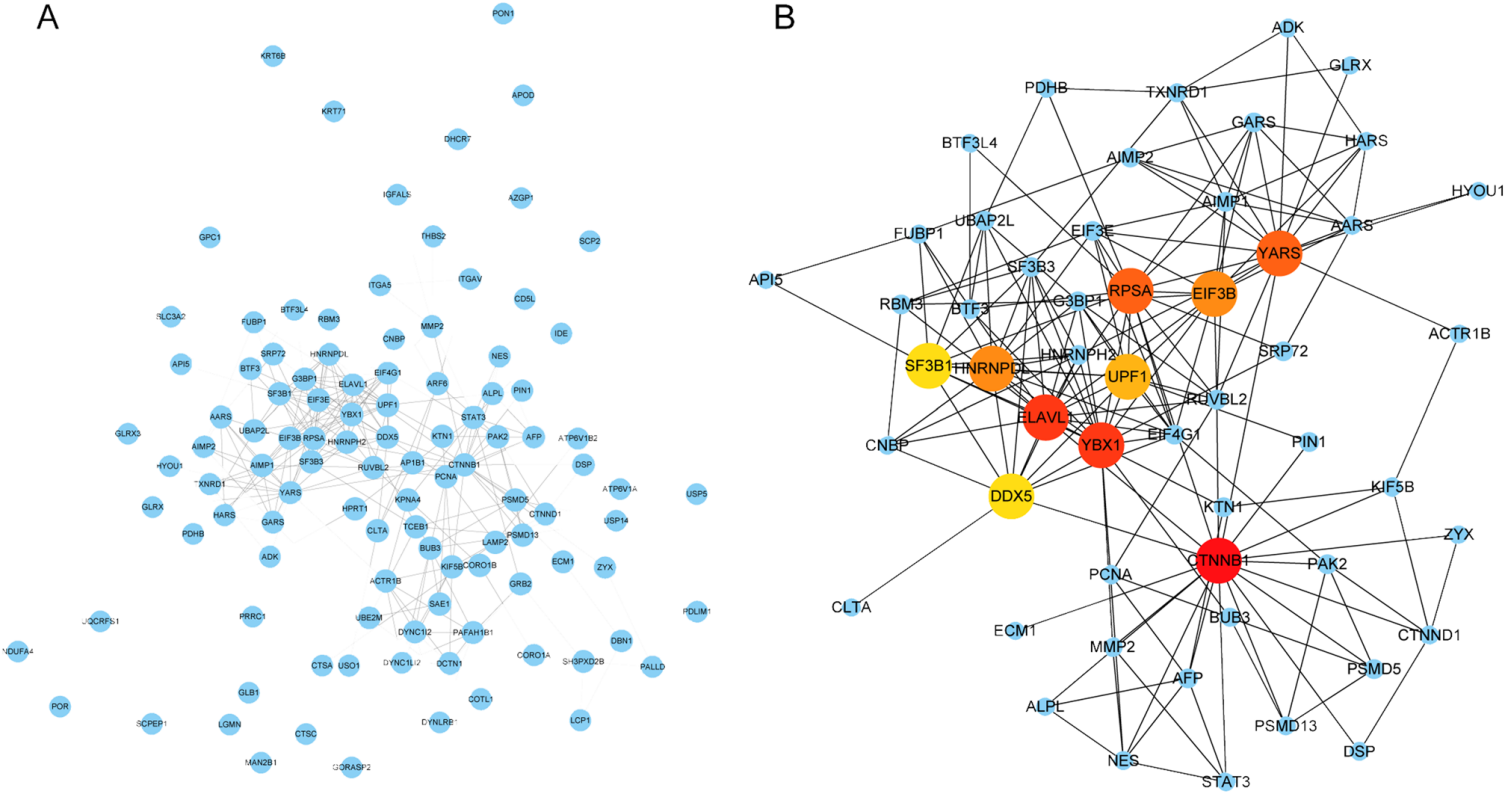
The PPI network for antler-special proteins constructed by STRING and Cytoscape. (**A**) The whole network including 107 nodes and 289 edges. The disconnected proteins in the network did not be showed. (**B**) Sub-network of top ten hub proteins with their expanded network.

The proteins detected in all samples can be considered as core proteins. In this experiment, 913 antler core proteins were detected accounting for 47.13% of the total identified proteins (Fig. 3A). To obtain a functional overview of these continuously expressed proteins, a gene ontology (GO) annotation enrichment analysis was performed. The core protein was mainly enriched in cell–cell adhesion, translation, Wnt signaling pathway and extracellular matrix organization (Fig. 3B, Table S3). Subsequently, the antler core proteins were compared with the costal cartilage proteins in order show the specificity of antler. A total of 132 antler core proteins was absent in the costal cartilage tissue (Fig. 3C). The network of predicted associations for 132 antler specific protein were constructed using STRING and Cytoscape (Fig. 4A). The top ten proteins ranked by degrees of the cytoHubba application in Cytoscape were recognized as hub proteins: CTNNB1, YBX1, ELAVL1, YARS, RPSA, EIF3B, HNRNPDL, UPF1, SF3B1, DDX5 (Fig. 4B).

### Analysis of stage-specific expression proteins

**Figure 5:**
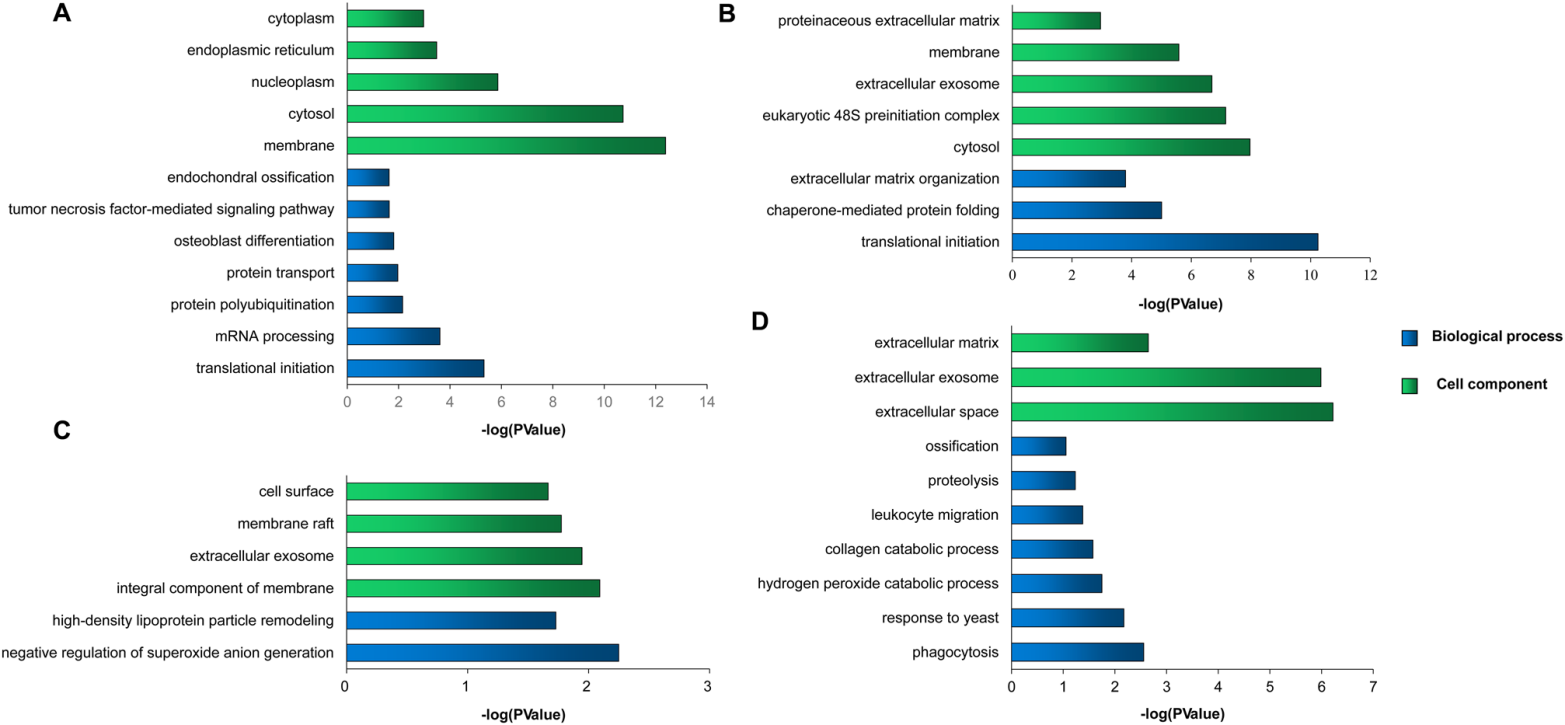
The GO enrichment result for antler stages-special proteins. (**A**–**D**) respectively represent the results of specific expressed proteins for the first four stages, the first five stages, 100 days and 130 days.

It is found that a total of 177 proteins were specifically expressed during the growth phase compared with the ossification phase (Fig. 3A). Among that, several proteins involved in chondrogenesis showed relatively higher LFQ intensity, such as collagen alpha-1(II) chain, hyaluronan and proteoglycan link protein 1, hyaluronan and proteoglycan link protein 3, bifunctional 3′-phosphoadenosine 5′-phosphosulfate synthase 1, bifunctional 3′-phosphoadenosine 5′-phosphosulfate synthase 2 (Table S1). The biological process enrichment results showed that these proteins were mainly involved in translational initiation, mRNA processing, Wnt signaling pathway, protein transport, osteoblast differentiation, endochondral ossification etc. Moreover, the cell component results showed that they were mainly localized in membrane, cytosol, nucleoplasm, endoplasmic reticulum etc. (Fig. 5A). 144 proteins were gone in the last time point of the ossification phase. Among that, aggrecan core protein is a major component of extracellular matrix of cartilagenous tissues and Collagen alpha-1(XI) chain may play an important role in fibrillogenesis by controlling lateral growth of collagen II fibrils. The enrichment results showed that these proteins were enriched into the biological process of translational initiation, chaperone-mediated protein folding and extracellular matrix organization (Fig. 5B). Although 100 days and 130 days were classified into ossification phase there were still significant differences. There were 33 proteins expressed specifically in 100 days. Among that, the LFQ intensity for keratin, type I cytoskeletal 19 is highest and it mainly is involved in the organization of myofibers. The second is Glutathione S-transferase A1, which is involved in the formation of glutathione conjugates of both prostaglandin A2 (PGA2) and prostaglandin J2 (PGJ2). Tartrate-resistant acid phosphatase type 5, a classical markers of bone resorption and osteoclast differentiation, is also concluded in the list of 100-day special protein. The enrichment results showed the proteins were mainly involved in negative regulation of superoxide anion generation and high-density lipoprotein particle remodeling and located in extracellular exosome (Fig. 5C). 53 proteins were screened as 130-day specifically expressed proteins. Among that, the immune response-related proteins were found with higher relative abundance, including azurocidin, neutrophil elastase, cathelicidin-7, myeloperoxidase and bactericidal permeability-increasing protein. Moreover, Matrix metalloproteinase-9, an important extracellular matrix degrading enzyme, is specifically expressed in 130-day antler. The enrichment results showed 130-day special proteins were mainly related with phagocytosis, response to yeast, collagen catabolic process, leukocyte migration and ossification and located in extracellular space, extracellular exosome and extracellular matrix (Fig. 5D).

### Analysis for significant differential expression protein

**Figure 6:**
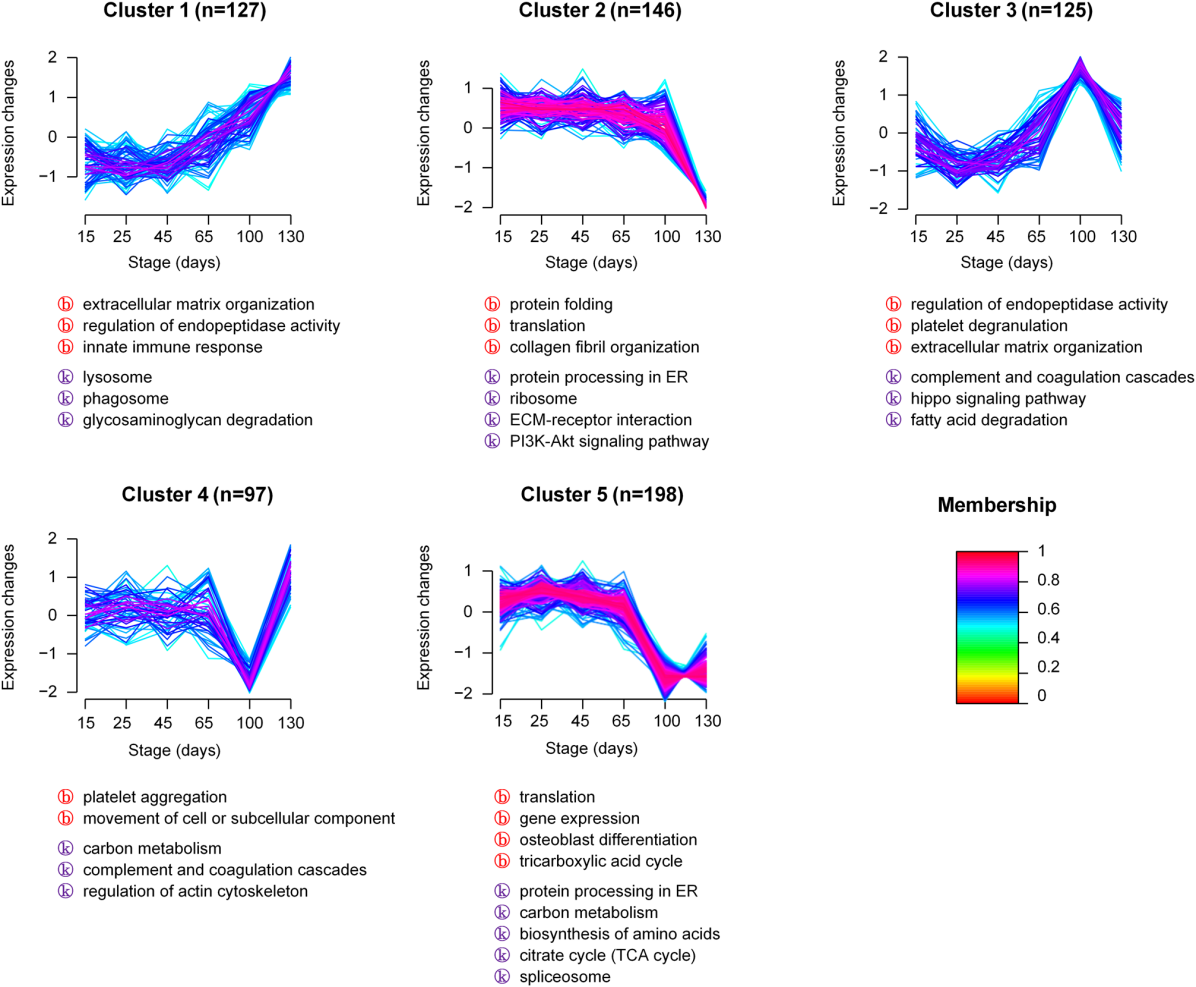
Global dynamics of proteins during antler development. The main function class was searched for GO (Biological process) and KEGG enrichment.

A total of 875 differentially expressed proteins were screened with *adjusted p-values* < 0.05 using one-way ANOVA (Table S4). We divided 693 DEPs proteins into five clusters according to the trends in the expression changes in the six different developmental stages by GProX clustering analysis (Fig. 6). Proteins with similar expression patterns were grouped together. The proteins related to Cluster 1 showed a relatively stable expression before 65 days and were up-regulated after that. In this category, extracellular matrix organization, lysosome and glycosaminoglycan degradation was a focus of special attention (Table S5). Cluster 2 proteins were down-regulated at 130 days. The proteins were mainly composed of gene expression regulatory proteins such as translation initiation, protein folding, protein transport. Cluster 3 showed a peak expression at 100 days including regulation of endopeptidase activity, platelet degranulation, extracellular matrix organization, fatty acid degradation etc. Cluster 4 decreased at 100 days and then showed a dramatic increase at 130 days including platelet aggregation and regulation of actin cytoskeleton. Cluster 5 contained the most DEPs and had a dramatic decline at 100 days and 130 days mainly involving translation, gene expression, TCA cycle and spliceosome.

## Discussion

Proteomics is a very useful method for studying biological development. In this study, we used label-free proteomics technology to identify the proteins of antler in the differential development stages and obtained a comprehensive proteomic data of sika deer antlers, containing 1937 proteins with high degree of credibility. Among these, 15 types of collagen were included, such as collagen I, II, III, IV, V, VI, VII, IX, X, XI, XII, XIV, XV, XVIII and XX. Collagen type II is a major matrix component of hyaline cartilage and collagen X is specifically expressed in hypertrophic zone in endochondral ossification. Besides, several bone proteins were also found in sika deer, such as osteopontin, bone sialoprotein (BSP), osteomodulin, SPARC. The data will provide valuable clues for understanding the molecular mechanisms of antler rapid growth and ossification.First, we analyzed the antler core proteins. Among that, 132 proteins were absent in costal cartilage and the to ten hub proteins were involved in gene expression regulation. CTNNB1 is a key downstream component of the classical Wnt signaling pathway, which plays a key role in promoting cartilage formation and bone formation during antler development^9,16,17^. Some studies have shown that CTNNB1 together with TCF proteins regulates osteoblast expression of osteoprotegerin, an important inhibitor of osteoclast differentiation^18^. The loss of CTNNB1 in osteoblasts leads to osteopenia and stabilization of CTNNB1 leads to high bone mass. The antler-special proteins indicated that antler had a unique developmental mechanism different from costal cartilage.

The hierarchical clustering results show that the antlers at 6 time points can be roughly divided into two stages, the growth phase (15, 25, 45 and 65 days) and the ossification phase (100 and 130 days). The antlers grow exponentially during the growth phase, but almost stagnates during the ossification phase^19^. Therefore, we screened the antler stage-specific proteins and significant differential expression protein. The results found that the expression pattern of these proteins has a distinct feature: in the growth phase, more protein expression is up-regulated. This unbalanced pattern has been revealed by previous quantitative proteomic analysis of cartilage formation^20^. Undifferentiated cells express mixed genes making the biological functions of these upregulated proteins more complicated compared with chondrogenic cells. Golan-Mashiach also reported that stem cells express promiscuous genes and have many differentiation options^21^. However, in the differentiated state, the expression of most genes decreases but expression of some specific genes increases. Therefore, the expression of cell protein may be related to its differentiation state.

In addition, collagen alpha-1(II) chain, collagen alpha-2(XI) chain and aggrecan, the main components of cartilage, were up-regulated in the growth phase of antler. Previous study showed that type II collagen was slightly expressed in young antlers (6 weeks) but absent in old antlers^22^. These proteins are required to maintain the normal physioligical function of cartilage. The deficiency will affect chondrocyte maturation, leading to the arrest of cartilage and long bone. Hyaluronan and proteoglycan link protein 1 (HAPLN1) with the highest relative abundance of special proteins for antler growth stages is also called cartilage-associated protein 1 (CRTL1). Hyaluronic acid is an important extracellular matrix in the process of cartilage formation. HAPLN1 stabilizes the aggregation of proteoglycan and hyaluronic acid by binding the hyaluronic acid chain in aggrecan. HAPLN1 may also be used as a growth factor to up-regulate the synthesis of aggrecan and type II collagen in cartilage^23^. Besides, two main enzymes were identified which are involved in the post-translational modification of chondrocyte ECM components, PAPSS1 and PAPSS2. In chondrocytes, the sulfation of proteoglycans is an important post-translational modification. In mammals, PAPSS plays an important role in the development of cartilage proteoglycan sulfation and adapts to the strong ECM synthesis during cartilage formation^24^. Therefore, it can be considered that chondrogenic differentiation may be the main characteristic of the rapid growth of antler.

The upregulated proteins during antler ossification were significantly enriched in lysosome, acidic membrane-bound organelle that contain proteins required for osteoclast bone resorptive function^25^. There is considerable evidence to suggest that Cathepsin B and cathepsin D are located in the lysosomes of osteoclasts^26–28^ and the inhibitors have marked inhibitory effect on bone resorption capacity^29^. Matrix metalloproteinases are a family of proteolytic enzymes that contribute to the degradation of the organic matrix of bones^30^. MMP9 is highly expressed in bone cells especially osteoclasts and regulates ECM degradation and bone remodeling. The MMP9 null mice showed improved connectivity density of the trabeculae^31^. The tartrate acid-resistant phosphatase (TRAP), which is commonly used as a specific marker of differentiated osteoclasts, is another lysosomal enzyme implicated in osteoclast resorptive function. It should be noted that the proteolytic enzymes generally require an acidic environment to be activated. Carbonic anhydrase II (CA II) is a zinc-containing metal enzyme that catalyzes the production of protons intracellularly from carbon dioxide^32^. These protons produced are transported by vacuolar H+-ATPases (V-ATPases) through the ruffled border cell membrane into the resorption lacuna^33^. ATP6V1A, a catalytic subunit of V-ATPases, is highly expressed in osteoclasts. Horng reported that knockdown of ATP6V1A can impairs acid secretion and ion balance in zebrafish^34^. The up-regulation of lysosomal-related proteins suggests that the bone remodeling activity of deer antler in the ossification stage is higher than that in the growth stage.

Bone growth and the immune system are believed to have a close interaction. Many immune factors affect the differentiation and bone resorption of osteoclasts by regulating the RANK/RANKL/OPG system such as tumor necrosis factor (TNF)-α, interleukin (IL)-1β, IL-6 and IL-8. In this study, some immune-related molecules, such as Protein S100-A7 (S100A7), cathelicidin-7 (CATHL7), lactotransferrin (LTF), azurocidin (AZU1), neutrophil elastase (ELANE) and myeloperoxidase (MPO), were found to be up-regulated during ossification. These immune-related molecules play an important role in the regulation of osteoblast and osteoclast differentiation. S100A7 promotes osteoclast differentiation and activity by enhancing the effects of M-CSF and RANKL^35–37^. However, several other factors effectively promote the proliferation and differentiation of osteoblasts and bone formation while inhibiting bone resorption^38–45^. SHAO also found that immune-related factors are related to the process of intervertebral disc ossification when analyzing the differentially expressed genes in herniated discs with or without calcification^46^. Therefore, we speculate that the local immune system may contribute to the ossification of antler tip.

Although we applied proteomics approach to obtain more important information about the development of sika deer antler, our study still has some limitations. In this study, we only performed 3 LC–MS/MS replicates to monitor the performance of the equipment, which is necessary for label-free proteomics approach. Only one biological “replicate” was used per time point, which may produce inconclusive results^47^. To lower the influence of inter-individual variation, we pay more attention to the change of protein expression between the growth period (15, 25, 45, 65 days) and ossification period (100, 130 days), and not the change between two specific time points, i.e. 25 and 45 days. From some animal tissue developmental proteomics research, we have also obtained some information that the changes in protein expression at different development stage seem to be greater than the inter-individual variation^48–51^. Therefore, the results of this study are still meaningful for discovering the molecular mechanism of rapid growth and ossification of antler, although we still need some follow-up experiments to verify some of the results in this study. Another limitation is the choice of protein extraction protocol. In this study, we used SDT buffer to extract antler proteins, which was efficient to extract hydrophobic proteins. However, the extraction of protein from bone tissue usually requires a decalcification step with EDTA or HCl, which will help us extract more extracellular matrix proteins.

In summary, this study used label-free proteomics to analyze the protein expression profiles of sika deer antlers in different developmental stages. This will provide valuable information for studying the molecular mechanism of antler rapid growth and ossification.

## Materials and methods

### Samples selection and preparation

3 4-year-old male sika deer (*Cervus nippon Temminck*) with the same father from a local deer farm (Changchun, China) were selected. Antlers were harvested at 15, 25, 45, 65, 100 and 130 days after casting of the previous hard antlers, respectively. Before that, the deer needed to be anesthetized by 5% xylazine at 0.5 mg/kg body weight intramuscularly, which was performed in accordance with the ARRIVE guidelines (https://arriveguidelines.org). The antlers were washed with 75% ethanol to remove contaminants from the surface. The growing tips were removed and cut into small pieces (1 cm × 1 cm × 1 cm). These pieces were immediately ground into power in liquid nitrogen and stored at − 80 °C for protein extraction. All animal experimental protocols were approved and authorized by the Chinese Academy of Agricultural Sciences Animal Care and Use Committee.

### Protein in-solution digestion

One gram of the raw powder was mixed with 5 mL SDT buffer (4% (w/v) SDS, 100 mM Tris/HCl pH 7.6, 0.1 M DTT). The sample was sonicated, then incubated in a periodic vortex ice bath for 2 h and centrifuged at 20,000 × *g* for 15 min at 4 °C to remove undissolved powder. The supernatant was collected and the protein solution was digested using Filter aided proteome preparation (FASP) method^46^. The peptides obtained by FASP were desalted with C18-SD Extraction Disk Cartridge. The lyophilized peptide was resuspended in 40 μL 0.1% formic acid solution.

### LC–MS/MS analysis

5 µL of sample containing 1 µg peptide was loaded onto an EASY-nLC 1000 system (Thermo Science) equipped with trap column (2 cm × 100 μm, 5 μm C18) and analytical column (Thermo Science, 10 cm × 75 μm, 3 μm, C18). Each sample was conducted with three 3 LC–MS/MS runs. Buffer A was water with 0.1% formic acid; Buffer B was 84% acetonitrile with 0.1% formic acid. The flow rate was 300 nL/min. The mobile phase and gradient were as follows: the linear gradient of the B solution ranged from 0 to 45% in 0–100 min; at 100–108 min, the linear gradient of the B liquid ranged from 45 to 100%; at 108–120 min, solution B was maintained at 100%; at 120–130 min, solution B was maintained at 5%. The peptide fragments were analyzed using Q-Exactive mass spectrometer (Thermo Science). The mass spectrometer instrument was operated in positive mode with a 1.85 kV applied spray voltage and drying gas flow of 4 L/min at 325 °C was used. The scanning range was 300–1800 m/*z*. First-order mass spectrometry resolution was 70,000 at 200 m/*z*, AGC (Automatic gain control) target was 1e6, maximum IT was 50 ms and dynamic exclusion was 60.0 s. The *m*/*z* of polypeptide and polypeptide fragments were collected as follows: 20 fragments were collected after each full scan, MS^2^ activation type was HCD, isolation window was 2 m/*z*, MS^2^ resolution was 17,500 at 200 m/*z*, normalized collision energy was 30 eV and underfill was set for 0.1%.

### Data analysis

The LC–MS/MS raw files were imported into MaxQuant software (version 1.5.3.17) for database retrieval. In this experiment, the protein database predicted using the combined methods of homology and de novo annotations with transcriptome and genome data of *Cervus nippon* (accession number GWHANOY00000000, https://bigd.big.ac.cn/gwh) were selected for retrieval and analysis. The database contains 21,449 protein sequences (Supplementary file 1). Relevant parameters settings are shown in Table 1. After the search was complete, the ‘proteingroups.txt’ output file was loaded in Perseus version 1.5.5.3. The proteins marked with reverse or potential contaminant were removed. The proteins identified with less than two unique peptides were also excluded from further analysis.

**Table 1:** Relevant parameters in MaxQuant software.

Item	Value
Enzyme	Trypsin
Max missed cleavages	2
Fixed modifications	Carbamidomethyl (C)
Variable modifications	Oxidation (M)
Main search	6 ppm
First search	20 ppm
MS/MS tolerance	20 ppm
Database pattern	Reverse
Include contaminants	True
protein FDR	≤ 0.01
Peptide FDR	≤ 0.01
Peptides used for protein quantification	Use razor and unique peptides
Time window (match between runs)	2 min
protein quantification	LFQ

### Bioinformatics analysis

Pearson correlation analysis were performed using the Perseus software. Upset plot and venn diagrams were drawn using TBtools (version 1.068). The network of protein–protein interaction was constructed by the online tool STRING (https://string-db.org/) with a filter condition (combined scores > 0.4) and the hub proteins were identified by degree using cytoHubba plugin in Cytoscape software (v3.8.0). The GO and KEGG pathway enrichment analyses were conducted based on DAVID Bioinformation Resources (version 6.8). The differentially expressed proteins for T15, T25, T45, T65, T100 and T130 were determined based on one-way ANOVA with adjusted p-values < 0.05. The GProX platform was used to cluster the differentially expression proteins with similar expression patterns. The number of clusters was set to 6 and a fixed regulation threshold (upper limit of 0.58 and lower limit of − 0.58) was used. The minimal membership for the plot was set as 0.5. Other parameters were set to default values.

### Ethical approval

The deer used in this study were approved and authorized by the Chinese Academy of Agricultural Sciences Animal Care and Use Committee. All experimental procedures were carried out in accordance with the approved guidelines and regulations.

## Supplementary Information

Supplementary Legend.

Supplementary Information.

Supplementary Tables.
