# Supplementary material for: Comparative antler proteome of sika deer from different developmental stages
Source: Sci Rep. 2021 May 18;11:10484. doi: 10.1038/s41598-021-89829-6 (PMC8131589; doi:10.1038/s41598-021-89829-6)
Supplement: Supplementary file 1 — Supplementary Legend. [file 41598_2021_89829_MOESM1_ESM.docx]

**Supplementary file: The .fasta text file for the proteome sequences**
